# Automated karyogram analysis for early detection of genetic and neurodegenerative disorders: a hybrid machine learning approach

**DOI:** 10.3389/fncom.2024.1525895

**Published:** 2025-01-22

**Authors:** Sumaira Tabassum, M. Jawad Khan, Javaid Iqbal, Asim Waris, M. Adeel Ijaz

**Affiliations:** Department of Robotics and Artificial Intelligence, School of Mechanical and Manufacturing Engineering, National University of Sciences and Technology, Islamabad, Pakistan

**Keywords:** chromosome anomalies, cognitive sciences, machine learning, neurological health, neurodevelopmental disorders, neurological disorders, neuroscience, genetic diseases

## Abstract

Anomalous chromosomes are the cause of genetic diseases such as cancer, Alzheimer's, Parkinson's, epilepsy, and autism. Karyotype analysis is the standard procedure for diagnosing genetic disorders. Identifying anomalies is often costly, time-consuming, heavily reliant on expert interpretation, and requires considerable manual effort. Efforts are being made to automate karyogram analysis. However, the unavailability of large datasets, particularly those including samples with chromosomal abnormalities, presents a significant challenge. The development of automated models requires extensive labeled and incredibly abnormal data to accurately identify and analyze abnormalities, which are difficult to obtain in sufficient quantities. Although the deep learning-based architecture has yielded state-of-the-art performance in medical image anomaly detection, it cannot be generalized well because of the lack of anomalous datasets. This study introduces a novel hybrid approach that combines unsupervised and supervised learning techniques to overcome the challenges of limited labeled data and scalability in chromosomal analysis. An Autoencoder-based system is initially trained with unlabeled data to identify chromosome patterns. It is fine-tuned on labeled data, followed by a classification step using a Convolutional Neural Network (CNN). A unique dataset of 234,259 chromosome images, including the training, validation, and test sets, was used. Marking a significant achievement in the scale of chromosomal analysis. The proposed hybrid system accurately detects structural anomalies in individual chromosome images, achieving 99.3% accuracy in classifying normal and abnormal chromosomes. We also used a structural similarity index measure and template matching to identify the part of the abnormal chromosome that differed from the normal one. This automated model has the potential to significantly contribute to the early detection and diagnosis of chromosome-related disorders that affect both genetic health and neurological behavior.

## 1 Introduction

**Figure 1:**
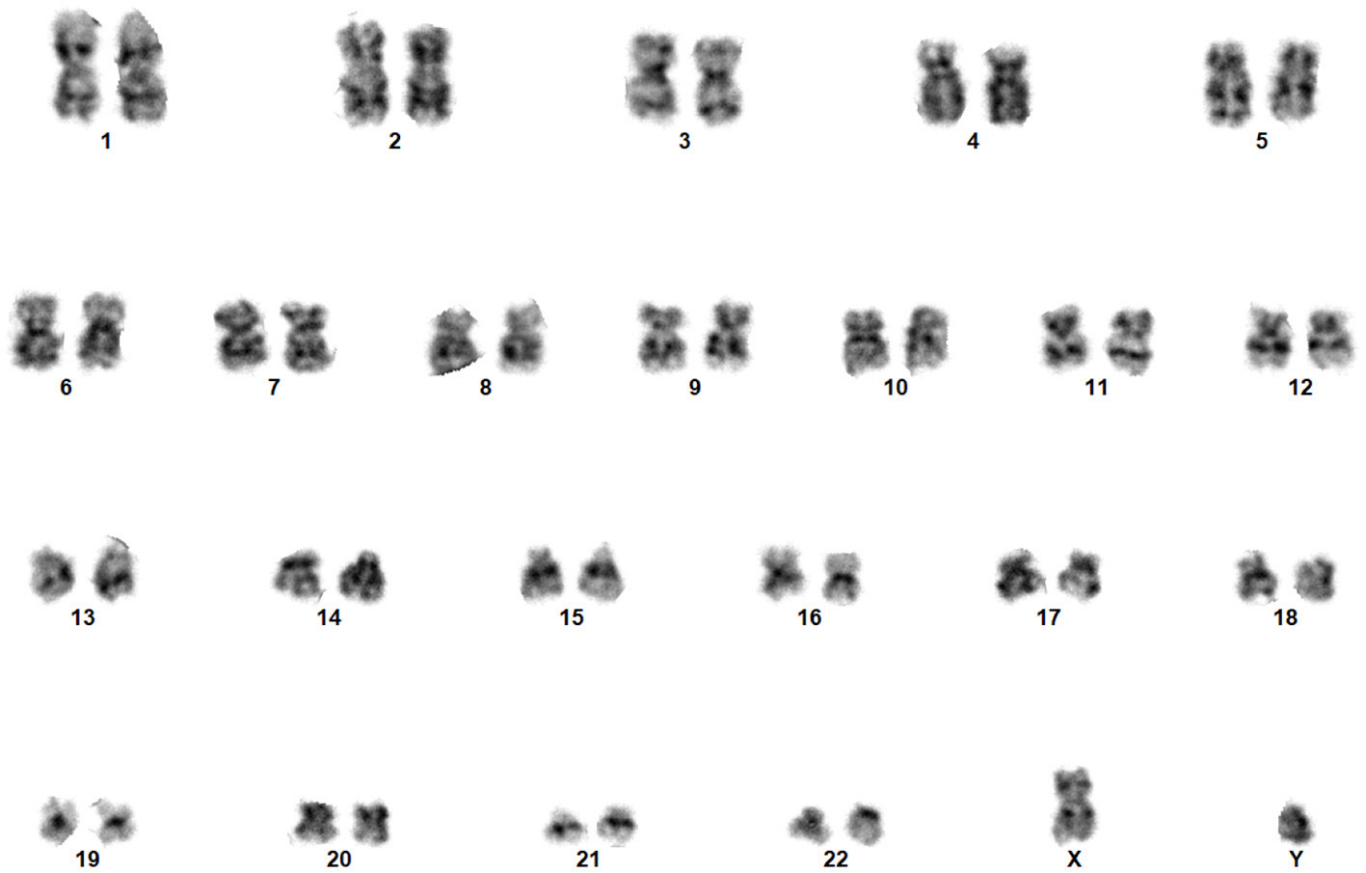
Normal karyogram of a typical human cell.

A chromosome is a thread-like structure that harbors genetic information encoded in genes. Located within the nuclei of cells in most living organisms, it comprises proteins and a solitary Deoxyribonucleic Acid (DNA) molecule. The structure of the chromosomes is shown in Figure 1. It transports genomic information from one cell to another (Institute, 2023). A typical human cell contains 46 chromosomes, comprising 22 pairs of single chromosomes (autosomes), which are numbered (1–22), and two sex chromosomes (XX or XY) (Institute, 2023). Chromosomes become visible during metaphase when stained with Giemsa and viewed under a light microscope. Understanding human chromosomes is crucial for diagnosing and predicting outcomes and tracking treatment progress under various conditions (Gersen, 2013). Cytogenetic experiments were performed to determine chromosomal abnormalities. Cytogenetics encompasses the examination of tissues, blood, bone marrow, and cultured cells i a laboratory setting. This field uses banding or manipulation techniques to identify chromosomal alterations (Natarajan, 2002).

Genetic diseases result directly from chromosomal abnormalities, and detecting chromosomal anomalies can anticipate and alert medical practitioners to potential diseases stemming from these abnormalities (Natarajan, 2002). Effective identification of chromosomal abnormalities is of significant clinical importance. Detecting genetic abnormalities in patients at the earliest stage is essential for timely and effective treatment. Chromosomal abnormalities are associated with genetic disorders. Changes in chromosome number or structure affect neurological health, such as Alzheimer's, Parkinson's, epilepsy, autism, and many other conditions. This can be detected using karyotyping. It is widely used for prenatal and fetal chromosome screening. The early detection of fetal chromosomal abnormalities can provide insights for detecting possible neurological and developmental abnormalities (Rosenfeld and Patel, 2017). Machine learning has been widely used in the detection of neurological disorders as it is used for the classification and segmentation of neurological images.

**Figure 2:**
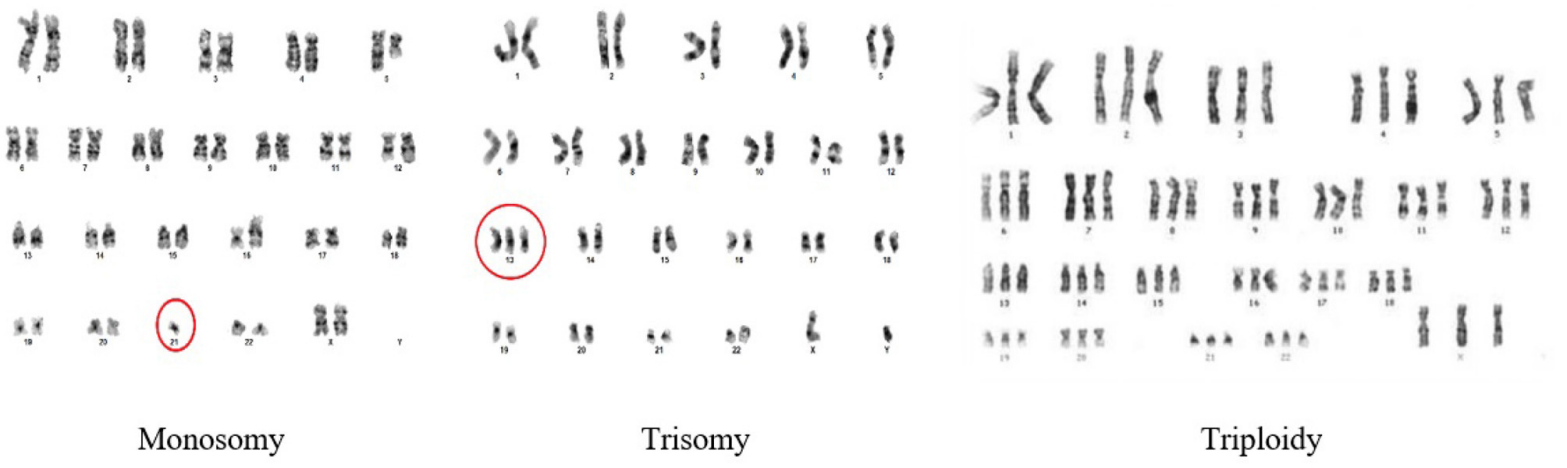
Numerical abnormalities in chromosomes.

Chromosomal disorders can be categorized into two primary types: numerical and structural abnormalities. A **numerical abnormality** signifies that an individual either lacks one of the chromosomes from a pair, or possesses more than two chromosomes instead of the usual pair. Numerical disorders arise from changes in the number of chromosomes, resulting in deviations from the expected count of 46. Examples of numerical disorders include trisomy, monosomy, and triploidy. Figure 2 shows the types of numerical abnormalities.

A **trisomy** occurs when a person has three of a particular chromosome instead of the usual two. Down Syndrome is caused by trisomy21. A **monosomy** occurs when they have just one chromosome instead of the usual two chromosomes. **Triploidy** is rare; however, in this type of abnormality, an extra third chromosome for each class is present in the cells.

**Figure 3:**
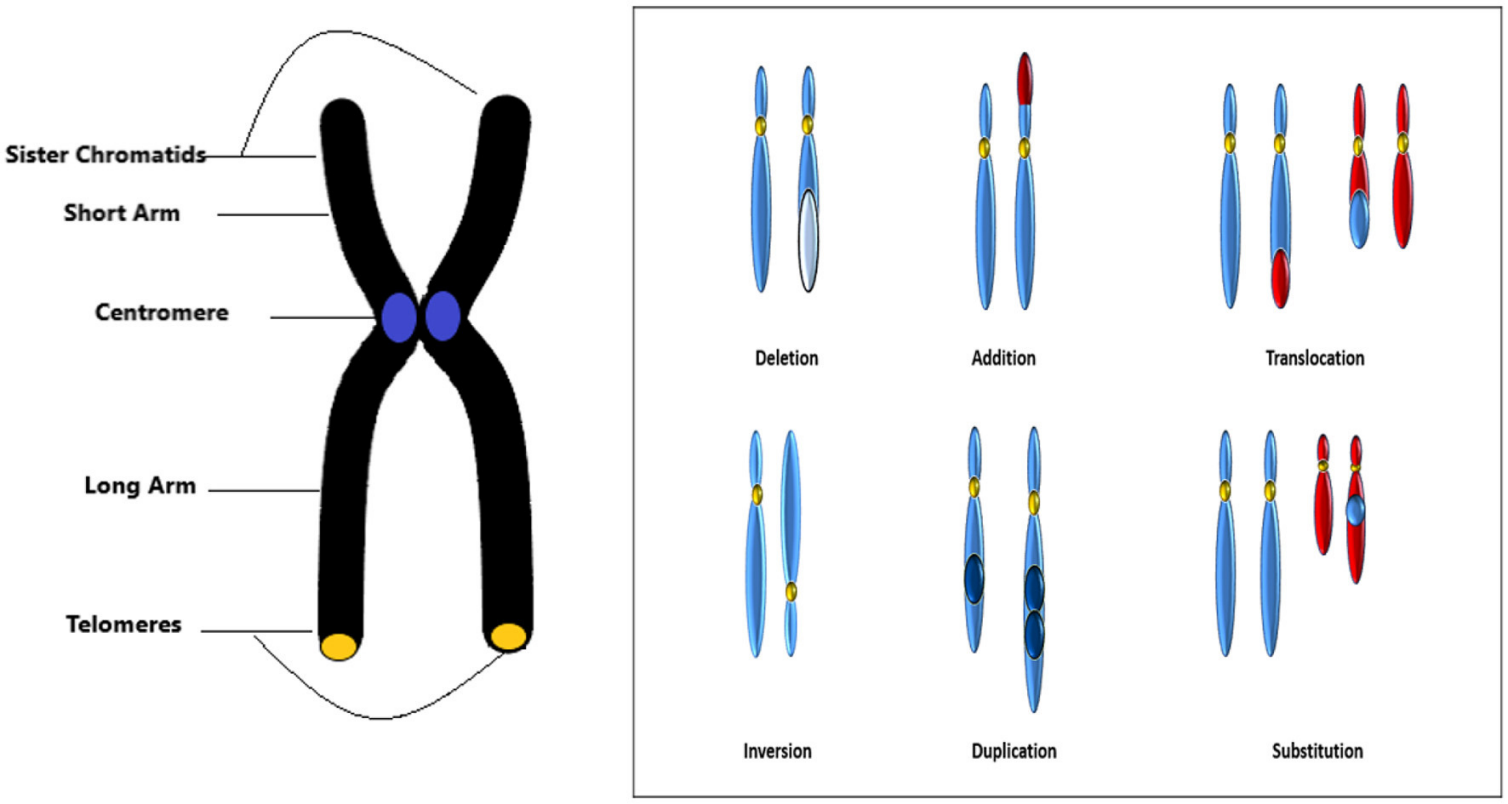
Chromosome structure and structural abnormalities.

Structural abnormalities indicate that the structure of the chromosome has been modified in various ways. Structural chromosomal disorders emerge from breakages within a chromosome or the incorrect rejoining of chromosomal segments. In such disorders, the number of copies of any given gene may exceed or fall short of two typical copies. Deletion, duplication, inversion, substitution, and translocation anomalies of the chromosomes are shown in Figure 3.

**Figure 4:**
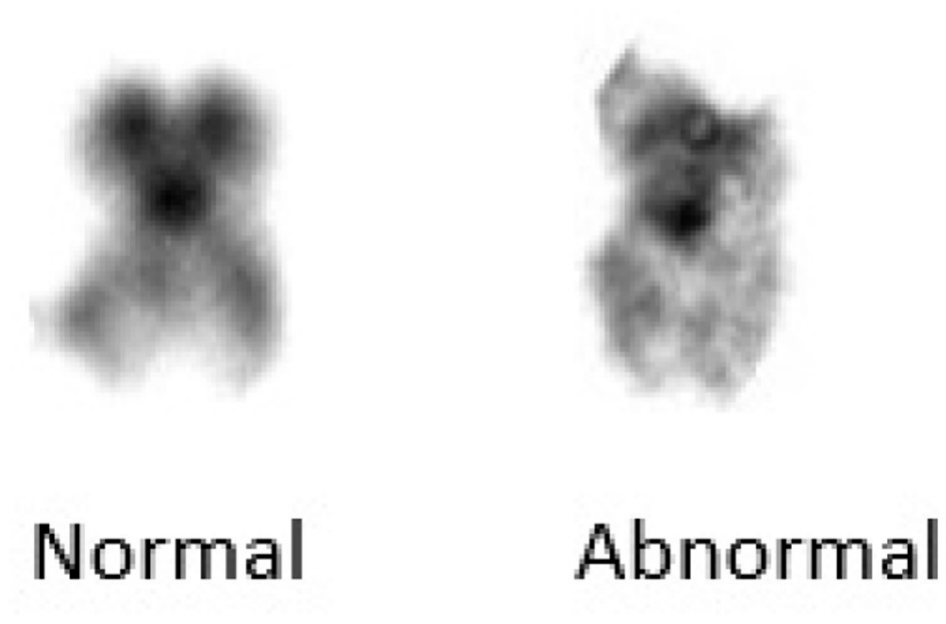
Chromosome 20.

Upon **deletion**, a chromosome segment is absent or deleted. This causes many abnormalities, for example deletion in chromosome 15 can cause angelman syndrome. In **duplication**, a portion of the chromosome is duplicated leading to excess genetic material like Dup15q Syndrome is caused by duplication of chromosome 15. In **inversion**, a chromosome segment may undergo problems such as breakage, can be turned upside down, and can have subsequent reattachment, causing inversion of the genetic material. **Substitution** occurs when a portion of a chromosome is replaced with a portion of another chromosome. **Translocation** appears when a part from one chromosome is moved to another. Translocation can be further divided into two types of **reciprocal translocation**, which occurs when segments from two distinct chromosomes have been interchanged, and **Robesonian translocation** occurs when an entire chromosome moves and fixes itself to another chromosome's centromere. In Figure 4, we show an example image of del20q chromosomes from our dataset.

Deletion, duplication, mutation, and trisomy are causes of cancer and neurological disorders such as epilepsy, Down syndrome, and autism spectrum disorder (ASD) syndrome. Neurological disorders are typically studied using electroencephalogram (EEG), ultrasonography, and magnetic resonance imaging (MRI). However, these techniques are usually applied after the onset of symptoms. These methods effectively monitor brain function once they are developed and visible. Genetic predispositions during the early developmental stages can be identified through chromosome analysis, which can help in the early diagnosis of such diseases. For this, fetal samples were collected and analyzed by karyotyping. This could help to identify any anomaly in chromosomes at the early stage of development, such as neurological disorders, before symptoms manifest. This way, karyotyping offers a more proactive approach to treatment and management.

### 1.1 Related work

Genetic diseases are mainly identified by karyotyping, but there are some diseases that different imaging techniques can identify. Methods commonly used for the detection of neurological disorders such as epilepsy often rely on EEG signals and various imaging techniques such as MRI. Machine learning has been used to automate the classification process of these techniques. Similar to multidomain feature fusion and selection approach proposed by Kong et al. (2024), it uses advanced signal processing and machine-learning techniques to optimize feature extraction and classification.

Machine learning (ML) has transformed healthcare by offering practical applications that have enhanced diagnosis, treatment, monitoring, and decision-making across various clinical domains. From the early detection of diseases to personalized treatment planning, automated reporting, and predictive analytics, ML models support healthcare practitioners in delivering more accurate, efficient, and scalable clinical solutions. This section outlines the key practical applications of ML in clinical workflows across different areas of healthcare, showcasing its versatility and impact beyond specialized fields like cytogenetics. For example, AI models are used in medical imaging to review X-rays, MRIs, and Computed Tomography scans to identify fractures, tumors, and organ failures as efficiently and accurately as possible.

Ibrahim et al. (2024) explored how deep learning using a pre-trained AlexNet model can help classify chest X-ray (CXR) images into four categories: COVID-19 pneumonia, non-COVID-19 viral pneumonia, bacterial pneumonia, and routine. Ahmad et al. (2024) introduced a computer-aided diagnosis (CAD) system for detecting breast cancer by combining deep learning and computer vision techniques. Islam et al. (2024) introduce BrainNet, a deep learning method for accurately classifying brain tumors using MRI images.

Montobbio et al. (2024) emphasized the potential and challenges of computational modeling and machine learning approaches for diagnosing and treating neurological disorders. Their insights, particularly in disease diagnosis, classification, and personalized therapeutic strategies, highlight the promising applications of these techniques. All of them used EEG and MRI images. Duarte et al. (2024) used flair images and machine learning for segmentation tasks. Alzheimer's disease (AD) was also diagnosed by Slimi et al. (2024) using machine learning on MRI images, and Li and Zhong (2024) explored the integration of deep learning in neuroscience, highlighting key trends and identifying major research hotspots in the field. Therefore, machine learning has been widely used for diagnosing such diseases but with different images adopted from different imaging techniques, as discussed earlier.

Anomaly detection by karyographic analysis is a common technique used to identify any numerical or structural abnormalities in human chromosomes. The conventional method for classifying chromosomes in most cytogenetic laboratories involves manual work by skilled experts. This procedure is time-consuming and requires significant effort from experienced operators, making it expensive. Experts commonly examine microscopic chromosome images in the conventional analysis of chromosomal anomalies, relying on their experience and expertise in detecting abnormalities that may lead to genetic disorders, congenital disabilities, or even cancer (Britto and Ravindran, 2007). The analysis of chromosome morphology involves a sequence of procedures, including selecting metaphase chromosome images. This encompasses the segmentation of individual chromosomes (Poletti et al., 2012), the classification of chromosomes (Madian et al., 2018), and the detection of chromosomal anomalies (Park et al., 2019). Significant efforts are being made to investigate how machine learning can improve pathological diagnosis. Deep learning technologies have experienced widespread adoption in recent years. The efficacy of these methods lies in their robust capacity for automatic feature extraction and learning from images, making them well-suited for the development of automated image analysis systems.

In medicine, artificial intelligence (AI) is being implemented, although some challenges exist. For example, the availability of labeled data is often limited, and labeling itself is challenging because of a lack of domain knowledge. Medical images containing anomalies are increasingly being analyzed using artificial intelligence. Aberrations, alternatively termed abnormalities, anomalies, or outliers, are often challenges in anomaly detection. The increasing popularity of deep learning-based anomaly detection algorithms is also facilitated by advancements in computational power and availability of big data.

Detecting aberrations poses a persistent challenge, particularly in the case of clonal chromosomal abnormalities in hematological malignancies. These abnormalities are characterized by their high complexity, diversity, and occasional rarity (Fang et al., 2023). To date, deep learning methods have been applied for detecting chromosomal abnormalities; however, challenges have arisen regarding data availability. Deep learning models rely heavily on data, and when it comes to the analysis of chromosomal aberrations, two primary issues emerge: privacy concerns and a limited amount of available data. Yan et al. (2019) employed ResNent to detect translocations between chromosomes 9 and 22 using only 200 individual karyotypes. Li et al. (2020) used generative adversarial network to detect anomalies in chromosome images using 320 images per class.

In this study, we attempted to automate the steps involved in detecting abnormal chromosomes in karyograms. Our approach involves feeding individual chromosomes into the model and identifying abnormal chromosomes. The primary contributions of this study are as follows:

1. We designed a hybrid deep learning model to identify abnormal chromosomes for genetic disorder identification.
2. We utilized unsupervised and supervised machine learning techniques to obtain the best results for classification.
3. We used a structural similarity index measure to distinguish the different parts of the anomalous chromosome from the normal one.
4. We performed template matching to identify the transloacted part of the abnormal chromosome.
5. We aimed to identify the most common structural abnormalities in neurological disorders by comparing the abnormal and normal chromosomes.

The remainder of this study is organized as follows:

Section II elaborates the proposed model for aberration detection for individual chromosomes. Section III describes the experiments and evaluation of model performance. In Section IV, we discuss the proposed method and its results. Finally, Section V concludes the study.

## 2 Materials and methods

### 2.1 Proposed approach

Our approach is Hybrid, combining both supervised and unsupervised methods. In this way, we are taking advantage of the small amount of labeled data available for anomaly detection. **Supervised learning** is a branch of machine learning, in which a model is trained using a labeled dataset. **Unsupervised learning** is a category of machine learning, in which an algorithm provides input data without specific instructions for processing it. This helps the model capture the underlying structure and variations in data.

**Figure 5:**
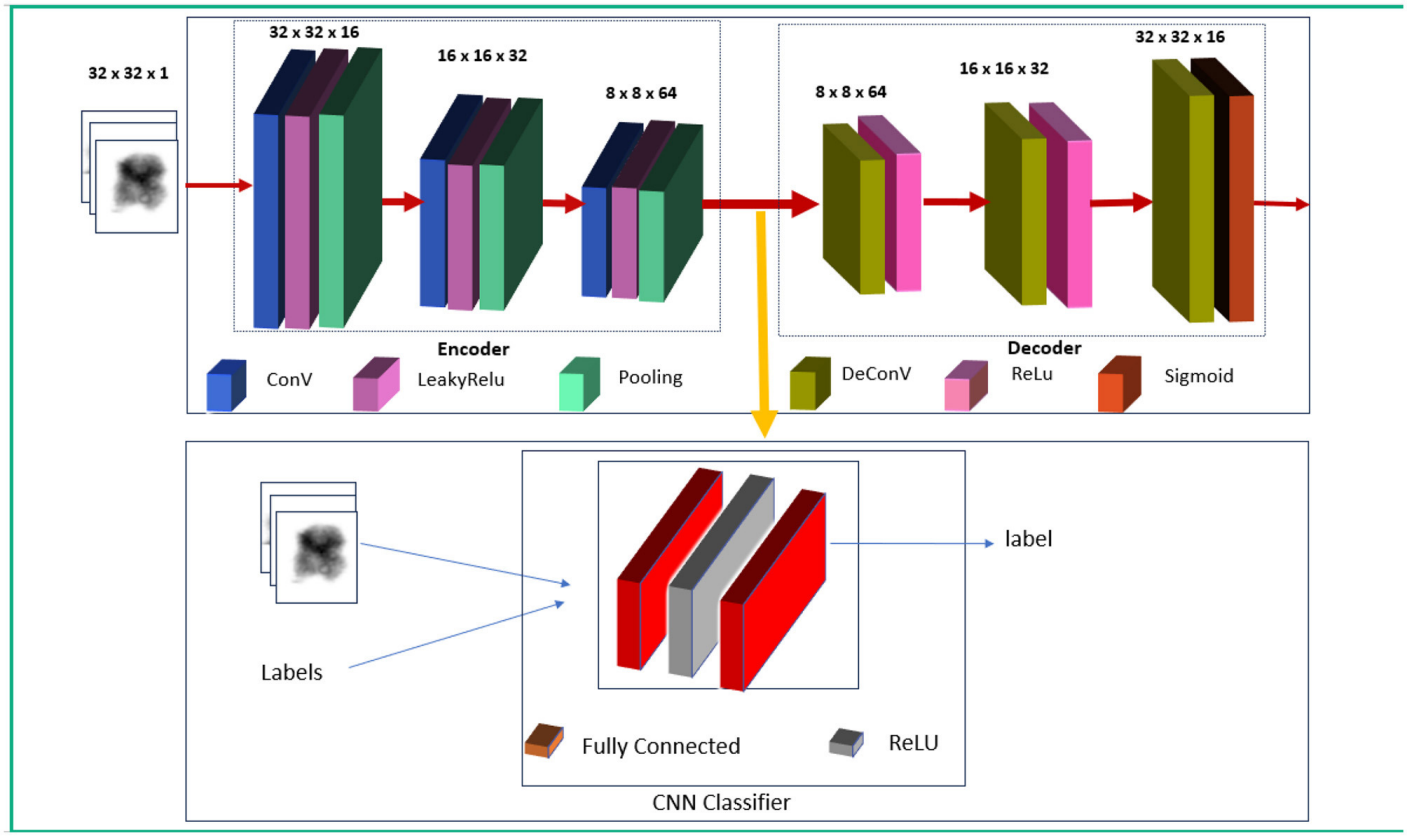
Proposed model.

The proposed system comprises of three major stages, as shown in Figure 5. The first stage involves training the autoencoder with unlabeled data. This is validated with both normal and abnormal data. The input to this stage is the individual chromosome extracted from the karyograms without labels. Chromosomes in the karyograms were arranged in classes. Therefore, we used karyogram singlets to determine whether the results were normal or abnormal. In the second stage, the encoder was utilized as a feature extractor. The extracted compressed features were fed into the CNN classifier as the input. Next, the CNN classifier is trained on the extracted features and labeled data. Finally, the encoder and classifier are trained using labeled data to fully leverage the encoder's ability to generalize from unlabeled data, enhancing its performance in classifying chromosomes.

### 2.2 Dataset

Images of chromosomes were used as a dataset that was manually annotated and verified by expert cytogeneticists. The dataset was divided into karyograms from which the individual chromosomes were extracted. In this study, we used images of singleton chromosomes for classification. Each chromosome was thoroughly inspected and annotated, and the final dataset of the individual chromosomes was verified by experts. The dataset comprises 234,259 individual chromosomes, of which 216,433 were normal chromosome images and 17,828 were abnormal chromosome images. This dataset included 7,412 chromosome images with translocation abnormalities and 10,416 chromosome images with deletion abnormalities. This ensures a comprehensive representation of the two anomalous categories. A total of 140,000 unlabeled normal chromosome images from all 24 classes were used to train the encoder, and 12,112 images including normal and abnormal chromosome images were used for validation purposes. The encoder and classifier were trained using 65,000 labeled chromosome images, of which 50,000 were normal chromosome images and 15,000 were abnormal chromosome images. To validate the encoder and classifier, we used 12,100 labeled chromosome images,including 1200 abnormal chromosome images and 10,900 normal chromosome images. A total of 5,047 chromosome were tested, including 426 abnormal chromosome images. Table 1 summarizes the distribution of the dataset.

**Table 1:** Summary of the dataset used for “training,” “validation,” and “testing,” with “normal” and “abnormal” chromosome breakdown.

**Dataset type**	**Number of chromosome images**	**Normal**	**Abnormal**
Training images (encoder)	140,000	140,000	—
Training images (encoder + classifier)	65,000	50,000	15,000
Validation images (encoder)	12,112	10,912	1,200
Validation images (encoder + classifier)	12,100	10,900	1,200
Test images	5,047	4,621	428

Deletion, addition, and translocation are the primary chromosomal anomalies. If the quality of an image is not good, then it is not easy to detect anomalies accurately, and banding patterns are the core to identify structural abnormalities; if the banding pattern is unclear, it is difficult to identify anomalies in the chromosome. Another problem that hurdles chromosomal anomalies is whether the chromosome is straight or curved. To avoid this, we selected straight and good-quality chromosome images for our approach.

### 2.3 Proposed method

We employed both supervised and unsupervised learning methods to develop a model for detecting chromosomal anomalies. The key steps of our approach are as follows:

#### 2.3.1 Unsupervised training using autoencoder

It involves autoencoder training with normal data to capture normal chromosome features.

**An autoencoder (AE)** represents an unsupervised machine learning approach utilizes a multilayered feed-forward neural network (Albahar and Binsawad, 2020). Information is input into the input layer and then passed through a series of hidden layers, making AE a straightforward feed-forward network. Each layer contains a variable number of nodes or neurons responsible for processing the input and generating the output. These nodes are distributed across different layers, each connected to all nodes in the preceding layers. The input and output layers both possess an identical number of nodes, denoted as “n,” because of the symmetric structure of the autoencoder, which aims to reconstruct the input on the output side. The predictions generated at each node, facilitated by the activation functions, are transmitted to consecutive layers. An autoencoder comprises two primary stages: Encoder and Decoder (Tan et al., 2019). We utilized this part because the autoencoder is trained solely on standard chromosome images without labels. This phase aims to help the encoder learn the typical patterns and structures found in the standard chromosome images. As the encoder model only sees normal data, it specializes in understanding and encoding these standard patterns into a compressed, lower-dimensional latent space representation. The decoder part attempts to reconstruct the input image from the latent-space representation, allowing the AE model to learn a good feature for the extraction process. For generalization, we validated it using abnormal and normal unlabeled chromosome data.

#### 2.3.2 Feature extraction from trained encoder

Once the AE is trained, the encoder extracts features from normal and abnormal chromosome images. The encoder provided feature representations for each image fed into the classifier. The features extracted from the encoder contain latent representations of the input chromosome images. These features are compressed and abstract forms of the original images, capturing the essential characteristics of the chromosomes while discarding less critical details. These features contain information, such as chromosome patterns, shapes, and structures.

#### 2.3.3 Training the (encoder + CNN classifier) with extracted features (supervised learning)

The features extracted by the AE encoder are then passed to the CNN classifier, which learns to classify images based on the encoder's output. This step uses the labeled data to train the classifier. The CNN classifier learns to distinguish between normal and abnormal chromosomes based on the features extracted from the encoder and is trained with the standard and abnormal labeled images while keeping the encoder weights fixed (frozen).

#### 2.3.4 Fine-tuning of encoder and classifier

In this case, the encoder's weights are unfrozen, and the encoder and classifier are fine-tuned using the labeled data. The last two layers of the encoder are fine-tuned. Training only the last two layers is computationally efficient and preserves the robust pretrained knowledge of the encoder's initial layers. This step is also impactful, because these layers represent higher-level abstract features of the input data. These features are closer to the final compressed representation and contain critical semantic information, making them crucial for adapting the model to new tasks or datasets. Fine-tuning these layers allows the model to adjust the high-level features to the new dataset without drastically altering the generalized low-level feature extraction learned earlier. Focusing on these layers halps us to reduce the risk of overfitting, as they retain generalized features, which is beneficial as our dataset is small.

**Figure 6:**
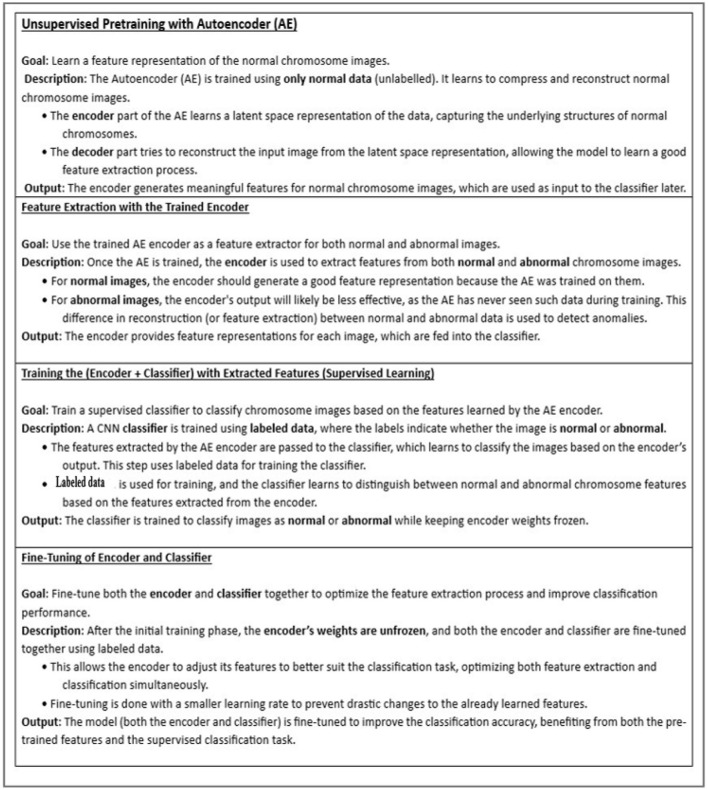
Flow of the proposed approach.

This step helps the encoder adjust its features to suit the classification task better. Simultaneously, the classifier learns to effectively map these extracted features to the desired classes (normal and abnormal chromosomes). By jointly optimizing both the encoder and classifier, the model can better capture discriminative features, improving overall classification accuracy. Finally, the model was validated using normal and abnormal chromosome images. The steps of our approach are shown in Figure 6.

The **encoder** plays a crucial role in our hybrid model, serving as the foundation for feature extraction and anomaly detection, enabling our approach to detect chromosomal abnormalities effectively. Its role can be broken down into several key functions:

1: Unsupervised feature extraction: The encoder is initially trained on unlabeled data, which then learns a compressed representation of chromosome images through an unsupervised approach. It then extracts meaningful latent features to capture essential chromosomal characteristics, such as patterns, shape, and structure. These features highlight important chromosome variations and anomalies, which are often difficult to detect using conventional methods.

2: Data compression and dimensionality: The encoder effectively performed dimensionality reduction by converting input chromosome images into a low-dimensional space. When non-essential information was discarded, only significant characteristics were preserved. This abstraction enriches the classifier by directing the implementation of the most essential features of the chromosomes, and enhances the general efficiency of the model.

3: Enhancing supervised learning of the CNN classifier: This extracted features are then given to the CNN classifier, which is trained on labeled data to differentiate normal chromosome patterns and abnormal patterns. The encoder output serves as a rich input representation, enabling the classifier to perform better by learning more discriminative patterns from these high-level informative features.

4: Fine-tuning for task optimization: In last stage, the encoder and CNN classifier are jointly fine-tuned with labeled data, enabling the encoder to refine its feature extraction process to suit the specific requirements of the classification task.

Therefore, this joint fine-tuning guarantees feature learning and classification in the best manner, thereby minimizing the generation of incorrect chromosomal anomaly detection models. It is worth noting that the encoder is a key component of the proposed hybrid model. It encompasses unsupervised anomaly detection to a supervised form of classification, allowing the system to deliver more accurate, scalable, and generalizable solutions to automate karyogram analysis.

#### 2.3.5 Anomaly detection

Once the hybrid model classifies chromosomes as abnormal, structural anomalies can be detected. For this purpose, we used SSIM and pattern matching to identify chromosomal abnormalities. The **SSIM** is a computer vision technique that identifies the differences between two images. It helps to identify the differences between chromosomes in cases of structural abnormalities, such as deletions, additions, and translocations. In the case of deletion or addition, the difference is clear; however, for translocation, we used the template matching technique. We first find the different parts from the normal with the help of SSIM. We also had to identify the translocated portion. For this purpose, we used pattern matching to find the translocated part. Pattern matching is a Computer Vision (CV) technique in which regions are located within an image that corresponds to the template. In this way, we successfully identified an anomalous part in chromosomes. Our main focus was to identify the structural abnormalities involving deletion and translocation in the chromosome structures.

a) Structural similarity index measure

SSIM was used to assess the quality of images by examining the structural details of two images (James et al., 2023).


(1) $$\begin{array}{l} \text{SSIM}=\frac{\left(2\mu_{x}\mu_{y}\right)\left(2\sigma_{xy}+c_{2}\right)}{\left(\mu_{x}^{2}+\mu_{y}^{2}+c_{1}\right)\left(\sigma_{x}^{2}+\sigma_{y}^{2}+c_{2}\right)} \end{array}$$

Where in Equation 1:

μ_*x*_ , μ_*y*_: Mean intensities of two images.

$\sigma_{x}^{2}$ + $\sigma_{y}^{2}$: Variances of intensities.

σ_*xy*_: Covariance of intensities.

*c*_1_,*c*_2_: Constants for avoiding instability when the denominator is close to zero.

**Figure 7:**
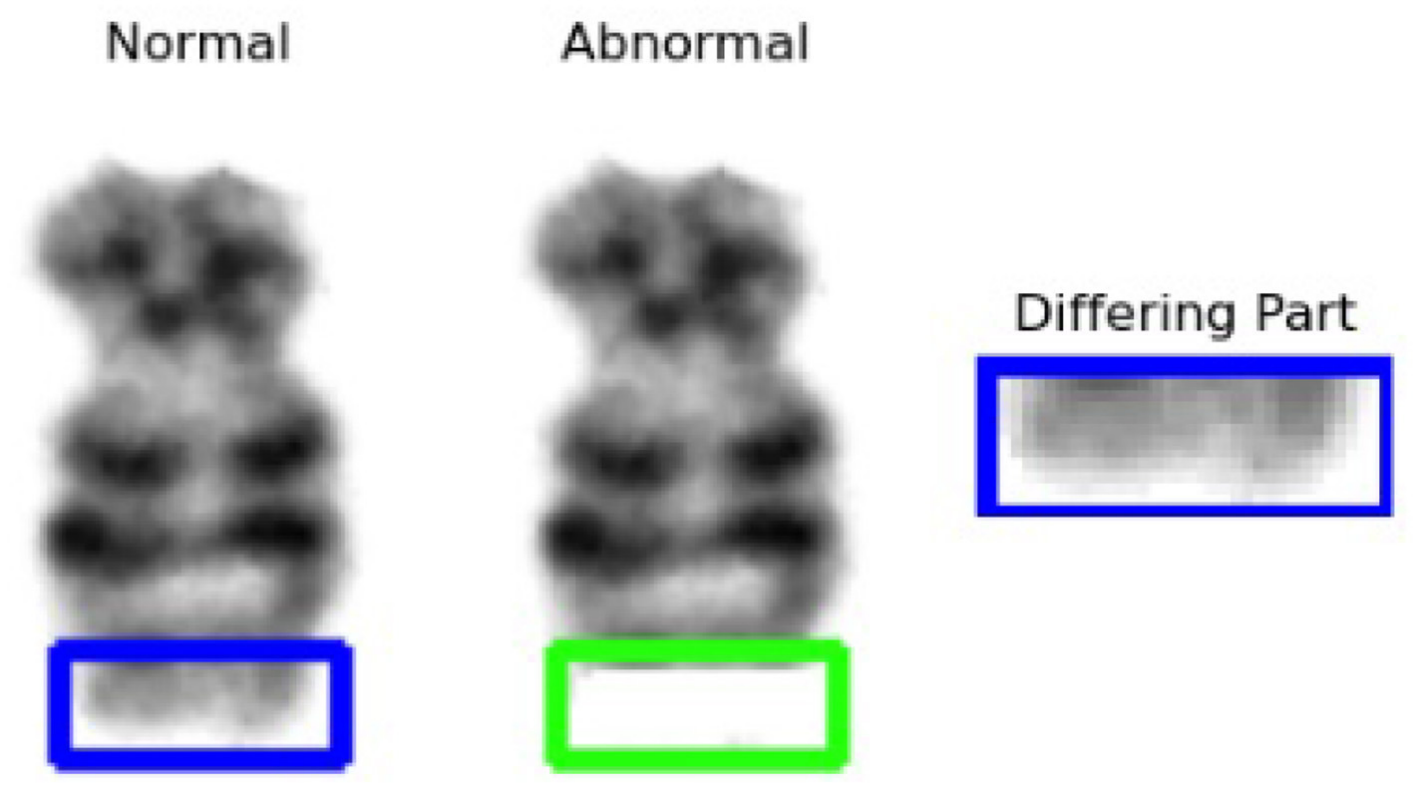
Structural similarity index measure result.

Figure 7 shows the implication of SSIM. b) Template matching:

Template matching is a machine-vision technique used to locate regions within an image corresponding to the template. A template is a predefined image or part of the image used to match the part in the main image. This process is performed by moving the template over the image. The similarity between the main image and the template image was calculated. Open CV template matching was then performed. The template image slides over the main image and the patch of the main image is compared with the template image.

## 3 Experiments and results

This section outlines the experimental setup, performance metrics, and the results of the proposed model.

### 3.1 Experimental setup

In this study, a CNN autoencoder and a CNN classifier were combined as models for classification tasks. Both models were trained using Python software. The Spyder platform (v. 5.4.3) was used for the training, validation, and testing of the model. The Spyder platform was implemented using PyTorch framework (v. 11.8 with torch version 2.3.0), and the experiments were conducted on UBUNTU 18.04, deployed with an NVIDIA RTX 1080 Ti.

### 3.2 Parameter setting and preprocessing

#### 3.2.1 Preprocessing of data

The images were preprocessed before being provided to the model as an input. Some of the images were large and some were small. The large images were compressed, and the small images were padded to obtain 32 × 32 dimensional images. This step was performed to maintain the uniformity of the images. We also normalized the images by scaling the pixel values to between 0 and 1.

#### 3.2.2 Parameters setting

**Figure 8:**
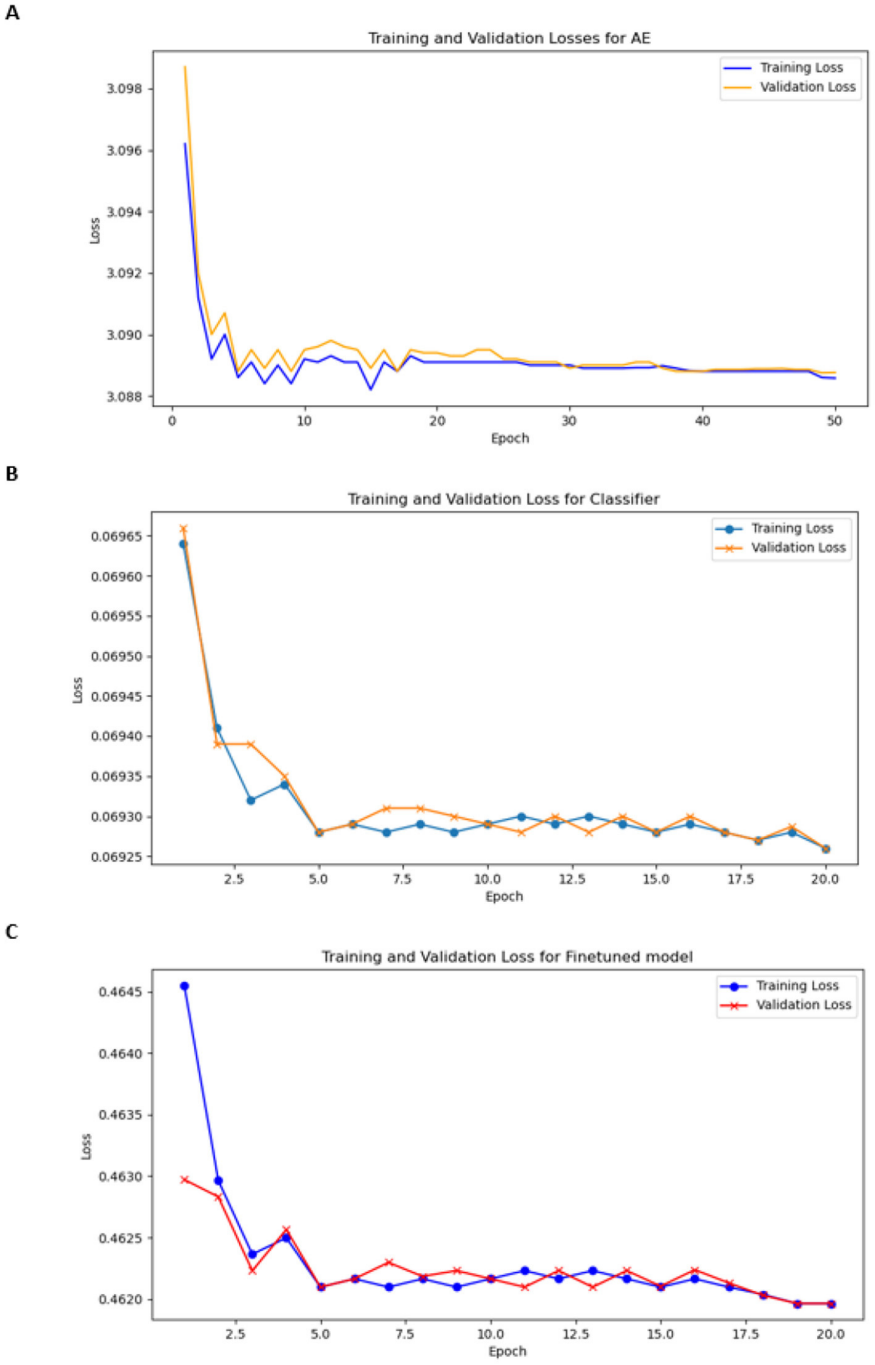
Training and validation losses **(A)** for encoder, **(B)** for classifier, and **(C)** for finetuned model.

- Encoder
  The encoder in our model consisted of three convolution layers with the following filter configurations: 16, 32, and 64. Each layer employs the Leaky ReLU activation function to enhance the learning of non-linear relationships and prevent vanishing gradient issues. The architecture progressively extracts hierarchical features from chromosome images, thereby capturing low- and high-level chromosomal patterns. We selected a batch size of 20 for training and 10 for validation. The optimizer was Adam, who had a learning rate of 0.001 and was trained for 50 epochs. We trained the model for 100 epochs earlier; therefore, the model could learn sufficient information in 50 epochs, so we stopped it at 50 epochs. The training and validation loss plots are shown in Figure 8A.

The loss function was MSE(Mean Square Error), as expressed in Equation 2.
**Mean squared error:**

(2) $$\text{MSE}=\frac{1}{n}\sum_{i=1}^{n}{\left(y_{i}-{\hat{y}}_{i}\right)}^{2}$$

Where
n: number of data points.
*y*_i_ : the actual value for the *i*^*th*^ data point.
$y_{\text{i}}^{ˆ}$: predicted value of the *i*^*th*^ data point.
This provides the mean of the squared discrepancies between the actual and predicted values, offering a metric for the overall accuracy of the prediction. For Normal data samples, the reconstruction error is typically low, whereas for anomalous data, the values tend to be higher and exceed a specific threshold.

- Decoder
  The decoder consists of three deconvolutional networks (deConvNets) with filter values (64,32,16) with ReLu.
- CNN classifier
  We selected a batch size of 20 for training and 10 for validation. Adam was used as the optimizer, with a learning rate of 0.0001, and was trained for 20 epochs. The loss function was CrossEntropyLoss. Figure 8B shows the training and validation losses.
- Encoder + classifier
  We selected a batch size of 20 for training and 10 for validation. The optimizer was Adam with the learning rate 0.0001 and was trained for 20 epochs. The loss function was weighted CrossEntropyLoss to effectively address the class imbalance, and the training and validation plots are shown in Figure 8C.

### 3.3 Model training

We implemented several strategies throughout the training pipeline to ensure the model's robustness and to mitigate overfitting and bias. The performance was continuously monitored on a separate validation set, and early stopping was applied based on validation loss trends to prevent overfitting, as well as regularization techniques such as the dropout layer. Data scaling was performed as an added data preprocessing technique, as it helped scale the input feature pixel values and achieve a stable convergence rate. To improve generalization, features were learned by passing both labeled and unlabeled data to the autoencoder before proceeding to the supervised classification component. Although the transformations used during data preprocessing did not include aggressive augmentation strategies such as flipping or cropping, we resized the chromosome images to a standard size of 32 × 32. Normalization was also applied to standardize the dataset's intensity range, ensuring sample consistency and minimizing noise.

Because the dataset was imbalanced, where abnormal chromosome samples were significantly fewer than the standard, steps were taken to prevent biased learning. Although the autoencoder was initially trained solely on standard samples to extract robust latent representations, the subsequent classifier was trained on normal and abnormal samples. For the evaluation, the test set comprised normal and abnormal chromosomes for a fair comparison of the model. Specifically, evaluation measures such as precision, recall, and F1-score for each class label were presented to measure the model's ability to identify deviations. Combined with this detailed evaluation and validation-based approach to monitoring during the training process, overfitting and accurate outcomes were significantly reduced.

### 3.4 Performance metrices

Four performance metrics were used for the evaluation. Accuracy was determined by dividing the number of correctly predicted cases by the total number of cases. A high accuracy value indicated that the model is made accurate predictions. Specifically, accuracy is calculated as the sum of true positives (TP) and true negatives (TN) divided by the total sum of true positives (TP), true negatives (TN), false positives (FP), and false negatives (FN), as shown in Equation 3.


(3) $$\begin{array}{l} \text{Accuracy}=\frac{TP+TN}{TP+FP+FN} \end{array}$$

Precision: Equation 4 measures the number of correct results out of all predicted positive results. It is calculated by dividing the number of true positives (TP) by the sum of true positives (TP) and false positives (FP).


(4) $$\begin{array}{l} \text{Precision}=\frac{TP}{TP+FP} \end{array}$$

Recall: This is also known as sensitivity or the true positive rate, which is the ratio of correctly predicted positive results to the total positive cases. It is calculated by dividing the number of true positives by the sum of the true positives and false negatives, as given in Equation 5.


(5) $$\begin{array}{l} \text{Recall}=\frac{TP}{TP+FN} \end{array}$$

F1 Score: The F1 score is the harmonic mean of precision and recall, providing a single metric that balances both. The Equation 6 helps calculate F1 score.


(6) $$\begin{array}{l} \text{F1 Score}=\frac{2\times \text{Precision}\times \text{Recall}}{\text{Precision}+\text{Recall}} \end{array}$$

### 3.5 Results

**Figure 9:**
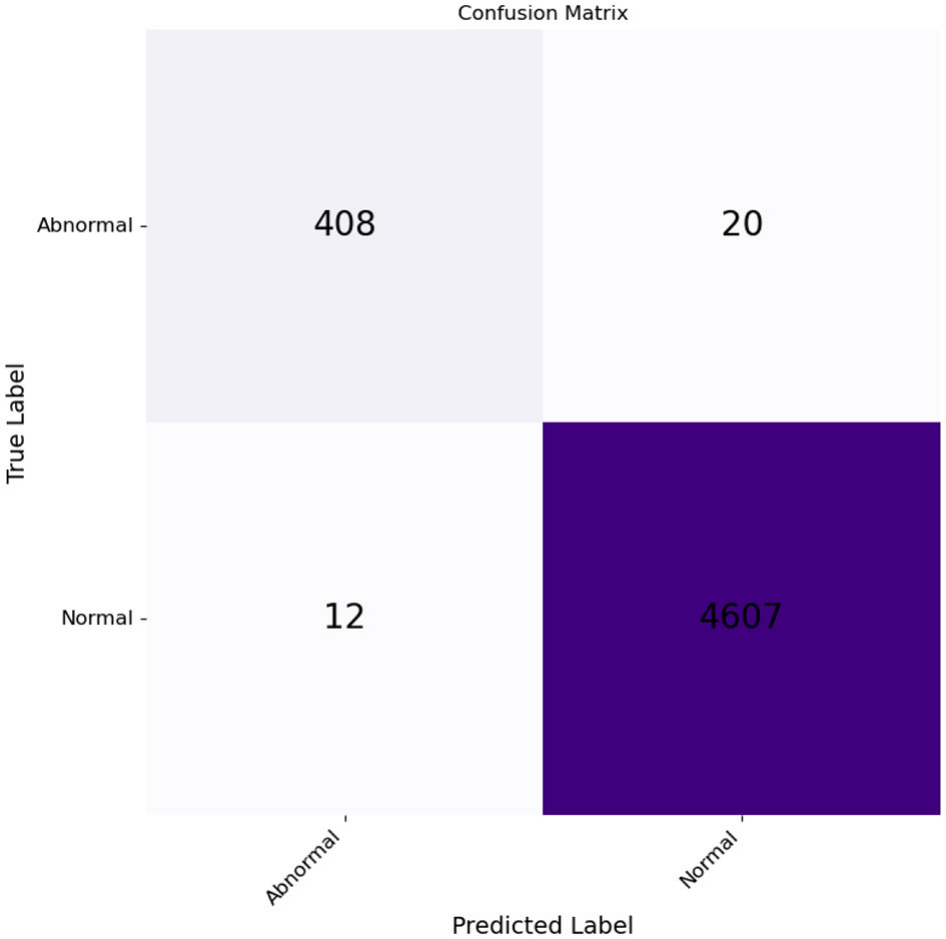
Confusion matrix for normal and abnormal classes plotted against the true and predicted classes.

The confusion matrix in Figure 9 shows the results. From the 428 input images of chromosomes, 408 were correctly classified as abnormal, and 20 were classified as normal. Of the 4,619 images of chromosomes, 4,607 were classified as normal and 12 were classified as normal, but were identified incorrectly as abnormal.

The evaluation metrics accuracy,precision, recall, and F1 score are summarized in Table 2 for our model.

**Table 2:** Model performance metrics.

**Metrics**	**Normal class %**	**Abnormal class %**
Accuracy	99.37	99.37
Precision	99.57	95.32
Recall	99.74	97.14
F1 score	99.65	96.22

**Figure 10:**
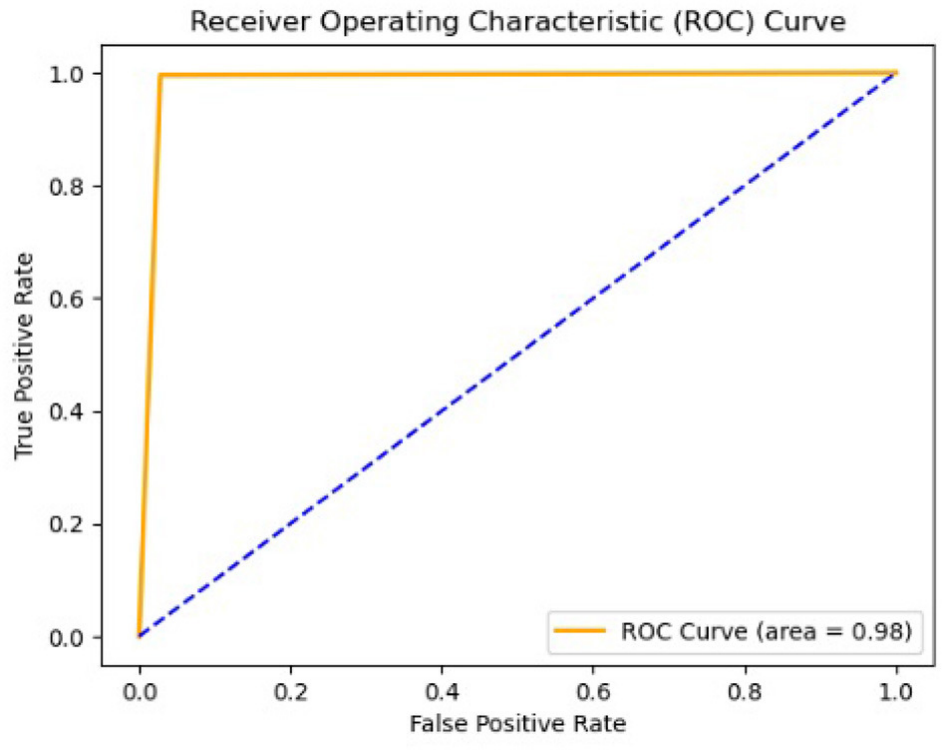
Receiver operating characteristic curve of the model.

A Receiver Operating Characteristic (ROC) curve was also generated to evaluate the performance of our model in predicting the probabilities of outcomes, distinguishing between normal and anomalous chromosome images, as shown in Figure 10. This curve was plotted against the true positive rate (TPR) and false positive rate (FPR). The area under the curve (AUC) was used to assess the level of discrimination between classes. Figure 10, with the value of AUC = 0.97, shows that our model is effectively distinguished between normal and abnormal chromosomes.

**Figure 11:**
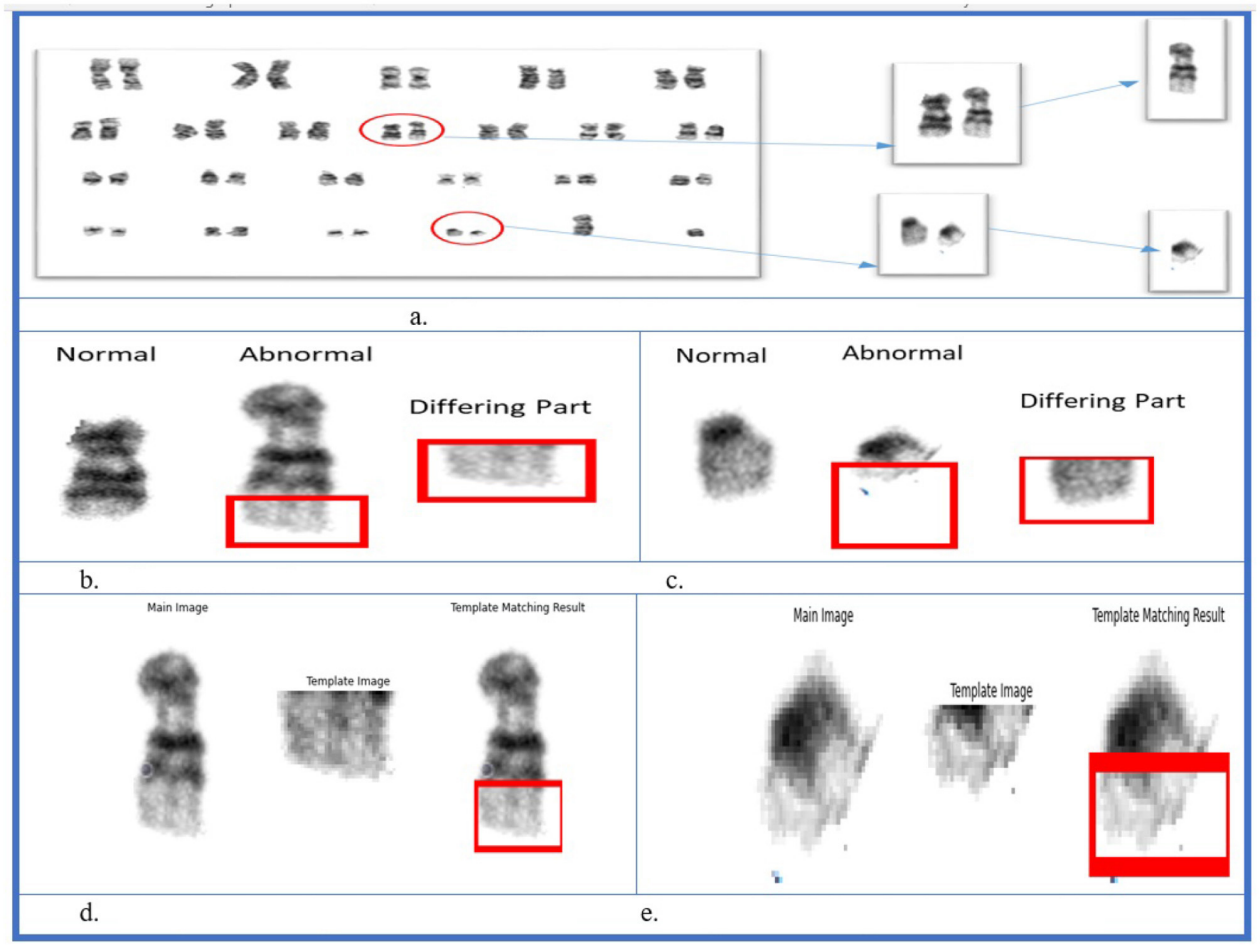
Structural similarity index measure and template matching results. **(A)** Karyogram with abnormal chromosomes. **(B)** Chromosome 9. **(C)** Chromosome 22. **(D)** Template matching for chromosome 22. **(E)** Template matching for chromosome 9.

In the dataset, only the translocations between chromosomes 9 and 22 were identified. Therefore, a pattern-matching technique was applied to detect abnormalities. As shown in Figure 11A, two abnormalities were observed in the karyograms: one on chromosome 9b and the other on chromosome 22b. Both 9b and 22b were identified as translocated chromosomes. In the first step, the two chromosomes were found to be abnormal. Subsequently, the type of abnormality was identified by comparing chromosomes 9 and 22 with their corresponding normal reference chromosomes. Differences between 9b and 22b were also observed. Different parts of chromosome 9 were identified using SSIM, as shown in Figure 11B. The same process was performed on chromosome 22, and different parts are shown in Figure 11C. In the final step, the template-matching technique was applied to locate the translocated parts. Figure 11D shows that part of chromosome 22 was located on chromosome 9, while Figure 11E shows the translocated part of chromosome 9 on chromosome 22. This approach enabled the identification of deleted or translocated parts of abnormal chromosomes.

## 4 Discussion

Cytogenetics is a branch of genetics that attempts to explain the relationship between human chromosomes and their genetic makeup and functions. Furthermore, it examines into the health and evolutionary implications arising from the architectural distortions of the chromosome. Cancer and other related abnormalities related to genetic diseases or neurological disorders are diagnosed after samples have been analyzed in laboratories. These methods are employed to search for and evaluate their effects, particularly on neurological disorders, in the health and developmental aspects of humans. This basic method of karyotyping is complex and requires a considerable amount of knowledge in the domain and time. Automated karyotyping enhances the speed and efficiency of chromosomal analysis, allowing for quicker identification of abnormalities. It reduces human involvement, addresses the challenges of manual analysis, and reduces the scarcity of large datasets. The major limitation observed is the absence of datasets because deep learning methods are data-intensive, and data related to abnormalities are much more complex and not easy to understand by every one.

Chromosomal analysis, when performed during fetal development, offers the unique advantage of detecting genetic abnormalities before the onset of clinical symptoms. This is crucial for disorders such as Down syndrome, autism, intellectual disabilities, edwards syndrome, cri-du-chat syndrome, mosaic Turner syndrome, and other underdevelopment disorders that have a strong genetic component. The earlier a disorder is detected, the earlier medical interventions, lifestyle adjustments, and support mechanisms can be implemented. Moreover, prenatal testing can allow families to prepare mentally and emotionally, while also making informed decisions about pregnancy, care, and future management of the child's health.

Disorders such as Down syndrome and other underdevelopment disorders are primarily identified and studied using techniques such as EEG, MRI, and other imaging technologies. However, these methods are only applied when a child or person shows signs of neurological disorder. For example, they can be identified when a child is already experiencing developmental delays or cognitive impairment. These technologies help monitor the brain's electrical activity and neural function. However, these studies do not offer predictive insights into the genetic basis of these conditions, particularly during the early stages of development. Visualizing chromosomes at an early stage allows the early detection of chromosomal abnormalities during fetal development. Anomalies such as deletions, duplications, or translocations that cause neurological disorders can be identified by analyzing fetal cell chromosomes.

With the advancement of deep learning models, including unsupervised and supervised approaches, it is now possible to automate and scale the analysis of chromosomal images of fetal or later blood or bone marrow samples. This automated analytical approach is more accurate and efficient. We introduce a hybrid model approach that utilizes unsupervised learning and supervised techniques. This hybrid model can efficiently process genetic data to quickly identify anomalies and provide more precise diagnoses. This facilitates the identification of structural abnormalities that are often associated with neurological disorders.

Our objective was to achieve the automatic detection of any structural chromosomal abnormality without the necessity for training for each distinct abnormality with labels. Our approach is beneficial because labeled examples are scarce, especially for rare anomalies. Prior CV and ML studies have addressed various challenges related to chromosomes (Boddupally and Thuraka, 2023), including segmentation, and Saleh et al. (2019) proposed Unet for chromosome segmentation. Fan et al. (2024) proposed DaCSeg for segmentation of chromosomes. Kang et al. (2024) proposed the model UC-Det model for counting chromosomes. Classifications: Qin et al. (2019) designed Verifocal-net for chromosome classification. Chang et al. (2024) proposed a DL model that uses attention to classify chromosomes. Wu et al. (2018) used GANs for the augmentation of chromosomes. Uzolas et al. (2022) used GANs for chromosome generation. Al-Kharraz et al. (2020) used YOLOV2 and VGG19 to identify the numerical aberrations. Wang et al. (2010) detected translocation in chromosomes using an adaptive matching technique. Kao et al. (2023) proposed 3 step process for identifying individual and clustered chromosomes. Cox et al. (2022) provided a supervised technique to identify abnormal chromosomes using Residual CNN. Bechar et al. (2023) used a supervised Siamese Network to classify chromosomes. Among the various studies mentioned previously, the prevalent approach involves the application of traditional supervised learning methods on relatively small datasets.

### 4.1 Significance of proposed approach

The proposed model integrates supervised and unsupervised learning techniques, leveraging the strengths of both approaches to improve the performance and robustness of automated chromosome classification.

#### 4.1.1 Supervised learning

Supervised learning uses labeled data to train models with the objective to learn a mapping between input features and their corresponding output labels. There are some **advantages** like: with a sufficient amount of labeled data this approach excels at learning discriminative patterns and distinguishing between normal and abnormal chromosomes with high accuracy. Supervised learning excels in tasks such as classification with high accuracy, particularly when labeled data are abundant. However, this is limited by the challenge of acquiring large labeled datasets in clinical settings. Supervised models also have some **disadvantages**, such as their dependence on large amounts of labeled data. Obtaining a large amount of labeled data requires significant time and expertise, which is a limitation in the clinical environment. and a model trained solely on limited labeled data reduces the generalization ability for unseen abnormal cases.

#### 4.1.2 Unsupervised learning

Unsupervised learning aims to identify structures inherent in data without using labeled learning information. The merits of unsupervised learning include that it works with large amounts of data that are not labeled and is easier to access than labeled data. It excels at discovering hidden patterns and relationships that can work well for feature extraction and feature space dimensionality reduction thereby enhancing the computational performance and generalization across diverse data. However, this method has some limitations. It has no direct relation to the target outputs, which makes it unsuitable for tasks involving exact quantitative predictions without further processing. However, the extracted features are more complex to analyze, and comparing their performance without a labeled dataset is challenging.

Unsupervised learning extracts meaningful features without relying on the labeled data. In our approach, an autoencoder is used for feature extraction, providing compressed representations of chromosome images. Unsupervised learning also has some **advantages** over supervised learning, such as the unsupervised approach enables to utilize a large number of unlabeled chromosome images that are more readily available and cost-effective to acquire. **Robust feature extraction:** The encoder captures essential structural and morphological information about chromosomes, making it possible to detect subtle patterns that are difficult to capture using supervised methods alone. **Better generalization:** Because the encoder was trained and validated on a large dataset, it can generalize better across different variations and imaging conditions. Like supervised models, they also have some **Disadvantages:** as: **Indirect labels:** While unsupervised models are good at feature extraction, they do not directly map to class labels and require subsequent integration with a supervised classifier. **Interpretability challenges:** Understanding the exact features extracted by the encoder can sometimes be less interpretable than supervised models, making it harder to explain specific clinical findings.

#### 4.1.3 Hybrid approach

By combining supervised and unsupervised techniques, our model leveraged the strengths of both approaches. Supervised learning excels in tasks that require labeled data, particularly in distinguishing between normal and abnormal chromosomes. However, acquiring large labeled datasets, particularly for rare anomalies, can be challenging. In contrast, unsupervised learning can handle large amounts of unlabeled data and is effective for feature extraction and pattern discovery. However, it lacks direct connections to target outputs, making it less suitable for classification tasks.

To address these limitations, our hybrid approach integrates the advantages of both methods. The unsupervised encoder extracts meaningful features, whereas the supervised classifier refines these features for the accurate classification of normal and abnormal chromosomes. This combination allowed us to harness the power of unsupervised learning for handling large unlabeled datasets and the precision of supervised learning for effective classification.

Table 3 compares our method with other methods, emphasizing the differences in the learning patterns. Our hybrid approach uses an autoencoder (AE) trained on unlabeled data for feature extraction, followed by a supervised classifier for the final classification task. Because normal data are often more abundant and easier to obtain than abnormal data, an autoencoder uses normal data to extract features. This eliminates the need for labels thereby allowing the autoencoder to autonomously identify valuable features from the dataset. The classifier then focuses on the most relevant features provided by the autoencoder thereby enhancing the classification performance. In addition, as the autoencoder is trained on unlabeled data, its reliance on labeled samples decreases, which is particularly beneficial when labeled anomalous data are scarce or costly.

**Table 3:** Comparison of our hybrid approach with existing approaches for karyogram analysis.

**Aspect**	**Existing models**	**Our approach (hybrid)**
Model architecture	CNN, Fully Connected Networks	AutoEncoder (Unsupervised) + CNN Classifier (Supervised)
Approach type	Mostly supervised	Hybrid (Supervised + Unsupervised)
Dataset diversity	Often limited to normal or simple anomalies	Comprehensive dataset with deletion and translocation structural abnormalities
Model generalizability	Poor generalizability on rare anomalies	Better generalizability as trained on unlabeled data

We trained, validated, and tested our model using a large data set that is not publicly available. The dataset contains not only normal chromosomes, but also abnormal chromosoems. After intensive training and validation, we tested our model on test data comprised of 5,047 images, including 428 abnormal images. Our model achieved an AUC value of 0.98, demonstrating its ability to distinguish between normal and abnormal chromosomes effectively. Our model outperforms identifying abnormal chromosomes from normal chromosomes using hybrid unsupervised and supervised deep learning. Compared to existing methods, as shown in Table 4, our hybrid approach achieved an accuracy of 99.3%, surpassing the DeepResidual model by Yan et al. (2019), which reached 97.5%, and the DNN model by Kang et al. (2024), which achieved 99.2% accuracy.

**Table 4:** Comparison with previous models.

**References**	**Model**	**Approach**	**Accuracy**
Yan et al. (2019)	DeepResidual	Supervised	97.5%.
Kang et al. (2024)	DNN	Supervised	99.2 %
Our approach	AutoEncoder + CNN classifier	Hybrid	99.3 %

Our approach comprises two distinct steps: first, detection of anomalous chromosomes, and second, identification of specific abnormalities within these chromosomes. The initial step was executed by employing normal images. We validated our AE model using a dataset containing both abnormal and normal samples. This demonstrates how our model is better than the others in detecting aberrant chromosomes; hence, we demonstrate our efficiency and precision in the hybrid mode.

After determining whether the chromosome is normal or abnormal, the following step seeks to determine a particular abnormality. Several methods in computer vision can detect abnormalities in chromosomes. Our approach involves aligning a normal chromosome with a counterpart chromosome to determine the area of the anomaly. Chromosomes are usually compared with normal chromosomes or ideograms to check for subtractive or translocation presence. To perform this task, we used the SSIM and pattern-matching methods. We compared the normal chromosomes instead of ideograms.

SSIM helps to identify the differences between the two images. We compared normal and abnormal images and identified different parts in cases of deletions and translocations. However, we first identified the difference between the normal tissue and different parts of the translocation. We also had to identify the translocated part and used pattern matching to find the translocated part. In this way, we successfully identified aberrations in the chromosomes. Our primary focus was identifying structural abnormalities involving deletion and translocation in chromosome structures.

We presented an approach for identifying structural aberrations in individual chromosomes extracted from karyograms. The methodology relies on analyzing banding patterns to detect and characterize these abnormalities. Substantial effort has been made to explore the integration of machine learning into pathology diagnoses. We presented a hybrid approach comprising both unsupervised and supervised learning that proved advantageous, particularly when dealing with a limited number of anomalous images. Gathering anomalous datasets in the medical field is inherently challenging. Our model was uniquely trained, validated, and tested on a large dataset, one of the first of its kind for this task, thereby significantly enhancing the robustness of anomaly detection and demonstrating its effectiveness in identifying chromosomal abnormalities.

In real-world scenarios, time constraints often lead to the standard practice of analyzing only a few meta-phase cells per specimen despite the availability of hundreds of cells. Despite this restricted analysis, challenges persist regarding the cost and turnaround time for diagnosis. This task is perfectly tailored for deep learning because of the complexity of expert analysis, which implies the use of visualization and the expected common mean of a sample set with its genus of origin. In addition, when applied to the initial assessment of chromosomal abnormalities for conditions such as epilepsy and Down syndrome, we expect that our model will provide prognostic advice for more effective patient management. The prediction of these disorders through the identification of genetic markers contributes to early intervention, which will help reduce the impact of the disorders on development as a result of early diagnosis and management. Recognizing neurological disorders at a preliminary stage significantly boosted genetic anomaly detection and preventive diagnostics in our model.

Here, we present a new methodology for a hybrid model to resolve the issue of automated chromosome anomaly detection, which is an important paradigm of cytogenetic analysis. Our study innovates by combining supervised and unsupervised learning frameworks, which enhances the detection accuracy and offers significant improvements over other methods. Our approach maximizes the value of the available data by utilizing unlabeled chromosome images during the feature extraction phase while still using labeled data for supervised classification. This strategy overcomes the limitations of imbalanced datasets, where obtaining many labeled abnormal chromosome images is difficult in clinical and research settings.

The method we propose for karyogram analysis is expected to greatly enhance the diagnostic process, allowing for the faster identification of potential genetic issues. Implementation of our model in clinical decision support systems can help cytogeneticists and practitioners obtain automatic and confident classification results, thus increasing diagnostic accuracy and reducing time.

### 4.2 Challenges in real-world adoption and limitations

In clinical contexts, it is essential to protect patient data. Simultaneously, datasets are usually associated with restrictions regarding the availability of information, which can be a problem for training and validation. In addition, the images of chromosomes in the model may not be consistent with those of other laboratories and imaging equipment, which might cause a difference. To handle such variations, robust domain adaptation techniques are necessary. Moreover, integrating our model seamlessly into existing laboratory software and clinical workflows requires technical compatibility and collaboration with various healthcare information technology (IT) systems. However, the proposed system has certain limitations. It is mainly used to work with straight chromosomes, but it is useful with curved chromosomes that are first straightened. However, refining the straightening process may enhance the outcomes. Although the model has shown promising results on the custom dataset used, it lacks validation on external datasets that contain similar complex structural chromosomal abnormalities, which could be an area for improvement. Despite its high classification accuracy, the model has significant computational demands, particularly during training. The concept of the model is defined by multiple convolutional layers that contribute to numerous parameters and significant GPU memory and processing power. This may restrict its application in the real world, especially in areas where resources are scarce, such as small clinical environments. However, a trade-off between computational inputs and model efficacy exists in the process. The inference times for such applications depend on the specific hardware, and in large-scale clinical trials, selecting the best hardware resources and their integration solutions is crucial. In our opinion, we can eliminate all of these problems with the help of further development of interdisciplinary cooperation, additional model refinements, and numerous clinical trials that will allow us to implement the proposed method in various clinics successfully.

## 5 Conclusion

Our study strongly emphasizes that reliable detection of anomalous data is important in medical applications, primarily in genetic diagnosis by karyotyping. Identification of anomalies in medical data is considered a task in computing science and is important for patient care and treatment. Therefore, developing robust methodologies such as the automated approach presented here is vital for ensuring the accuracy of diagnostic procedures. Our hybrid model, which combines an unsupervised encoder trained with unlabeled normal data and a supervised CNN classifier trained on labeled normal and abnormal chromosome data, is a powerful approach to karyogram analysis. Thus, by training the encoder with data that are not labeled as normal or abnormal and validating the model with normal and abnormal data, we ensure that we obtain the best of both worlds from a model where all the relevant features are captured. The encoder also learns the basic features that help enhance the task of chromosome classification; separating normal and abnormal chromosomes is performed accurately.

Our model was trained and validated using a large dataset, and eliminated false or misleading anomalies. Furthermore, we identified the anomalous chromosomes in detail using CV methods, SSIM, and template matching. Thus, the combined use of appropriate methodologies strengthened our approach and increased the accuracy of the results. After evaluating the test data, we found that precision, recall, F1 score, and accuracy were all impressive, with a total accuracy of 99.37% for both normal and abnormal classes, and F1 scores for both normal and abnormal classes were 99.65% and 96.22%, respectively. These results demonstrate that the model effectively classifies normal and abnormal chromosomes. In addition, the model achieved an AUC of 0.98, demonstrating its effectiveness in classifying normal and abnormal chromosomes.

Our study addresses the demand for automation in genetic disorder assessment and underscores the transformative potential of interdisciplinary approaches in healthcare and neurological computations. Future study will involve working with research laboratories and hospitals to obtain data on various imaging sources, lighting conditions, and types of chromosomal abnormalities. Moreover, while the system is currently designed to detect only structural anomalies, future study plans will incorporate numerical anomaly detection. We also plan to integrate explainable AI (XAI) to visually discuss the prediction results so that cytogeneticists and doctors can use this information efficiently for further case analysis.

## Data Availability

The data analyzed in this study is subject to the following licenses/restrictions: the dataset used in this study is proprietary and belongs to NUST. Due to ongoing research and confidentiality agreements, the data is not publicly available. However, interested researchers may contact the author for potential collaboration or for inquiries regarding data access within the constraints of institutional guidelines. Requests to access these datasets should be directed to tabassum.sumairaawan@gmail.com.
